# Quantitative Index and Abnormal Alarm Strategy Using Sensor-Dependent Vibration Data for Blade Crack Identification in Centrifugal Booster Fans

**DOI:** 10.3390/s16050632

**Published:** 2016-05-09

**Authors:** Jinglong Chen, Hailiang Sun, Shuai Wang, Zhengjia He

**Affiliations:** 1 State Key Laboratory for Manufacturing and Systems Engineering, Xi’an Jiaotong University, Xi’an 710049, China; wangsh_106551@yeah.net (S.W.); hzjvip123@163.com (Z.H.); 2 Beijing Institute of Astronautical Systems Engineering, Beijing 100076, China; hailiang41@live.cn

**Keywords:** fault diagnosis, blade crack, vibration signal analysis, quantitative identification, centrifugal booster fan

## Abstract

Centrifugal booster fans are important equipment used to recover blast furnace gas (BFG) for generating electricity, but blade crack faults (BCFs) in centrifugal booster fans can lead to unscheduled breakdowns and potentially serious accidents, so in this work quantitative fault identification and an abnormal alarm strategy based on acquired historical sensor-dependent vibration data is proposed for implementing condition-based maintenance for this type of equipment. Firstly, three group dependent sensors are installed to acquire running condition data. Then a discrete spectrum interpolation method and short time Fourier transform (STFT) are applied to preliminarily identify the running data in the sensor-dependent vibration data. As a result a quantitative identification and abnormal alarm strategy based on compound indexes including the largest Lyapunov exponent and relative energy ratio at the second harmonic frequency component is proposed. Then for validation the proposed blade crack quantitative identification and abnormality alarm strategy is applied to analyze acquired experimental data for centrifugal booster fans and it has successfully identified incipient blade crack faults. In addition, the related mathematical modelling work is also introduced to investigate the effects of mistuning and cracks on the vibration features of centrifugal impellers and to explore effective techniques for crack detection.

## 1. Introduction

Blast furnace gas (BFG) is a byproduct of iron-making. With the great expansion of the iron and steel industry, the production of BFG during iron-making has increased remarkably [1]. However, blast furnace gas is characterized in low calorific value, difficulty to burn and combustion instability as a power fuel [2], so how to deal effectively with blast furnace gas is a problem that puzzles iron and steel enterprises. In recent years, many enterprises have taken to use BFG to generate electricity in order to save energy and improve benefits. Centrifugal booster fans are important pieces of equipment which are used to pressurize BGF and make sure it can go into the furnace safely and combust stably. Faults occurring on the centrifugal booster fan may lead to accidents such as unstable combustion in the furnace, blow outs, downtime, and potentially huge economic losses.

Blade crack faults (BCFs) are among the typical faults in centrifugal booster fans. Different cracks arise after long running due to the resonance, decreased anti-fatigue capability because of manufacture problems, installation issues or the work conditions [3]. This may result in blades breaking off and the unit being damaged. For a rotor system, the stiffness of the shaft would display cyclical behavior once blade cracks occur [4], and the dynamic response signal will show obvious non-stationary characteristics. Fault diagnosis for blade crack faults is a difficult problem that has drawn the attention of top scholars and outstanding engineers worldwide [5,6,7,8]. Lots of related studies have been reported in important journals [9,10,11]. These researches place emphasis on the following aspects: first, the method based on finite element tries to model the cracked rotor and analyze the fracture mechanisms and the effect on the structural dynamic properties [12,13,14]. This method can provide a theoretical basis for the blade damage identification method based on vibration signals. Second, the method combining the finite element modeling with the signal processing makes use of the finite element method to analyze the dynamic responses of the cracked rotor [15]. Then a signal processing method such as the wavelet transform is applied to analyze the dynamic response and lock the position and size of the crack damage. However, the practical engineering of these methods, especially for blade crack fault detection under operation conditions, should be studied further. On-line and off-line condition monitoring systems have been widely used for important rotating machinery [16,17,18,19,20]. Vibration signals can be acquired effectively in various industrial fields [21,22,23,24]. The key issue is how to extract the characteristics of the blade crack faults using appropriate signal analysis methods [25].

In this paper, operation condition information is monitored and collected in a timely way based on a condition monitoring and fault diagnosis system for centrifugal booster fans. Firstly, aiming at the problem of energy leak in FFT, amplitude and phase can be accurately estimated by an discrete spectrum interpolation method [26,27]. The short time Fourier transform (STFT) can effectively pick out the non-stationary components in vibration signals [28]. The discrete spectrum interpolation method and STFT are integrated in the testing system. They are used to extract the features of the blade cracks of generator centrifugal booster fans in a certain iron and steel group’s power plants. Finally, for the purpose of quantitative identification and abnormality alarming for blade crack faults, a quantitative identification and abnormality alarm strategy based on compound indexes including the largest Lyapunov exponent (LLE) [29,30] and relative energy ratio at the second harmonic frequency component is proposed in this paper. The proposed method is applied to an accident caused by blade crack faults using historical data. The results demonstrate that this method could quantitatively identify blade cracks in booster fans successfully. In addition, related work on mathematical modelling is also introduced to investigate the effects of mistuning and cracks on the vibration features of centrifugal impellers and to explore effective techniques for crack detection.

The rest of the paper is organized as follows: in Section 2, the fault identification method for thermal generator sets is introduced. In Section 3, a case study via blade crack faults of centrifugal booster fans is presented. In Section 4, a quantitative identification and abnormality alarm strategy for blade crack faults is proposed. In Section 5, mathematical modelling for revealing vibration signal properties is introduced. In Section 6, some conclusions are provided.

## 2. Fault Identification Method for Thermal Generator Sets

### 2.1. Centrifugal Booster Fans in Thermal Generator Sets

**Figure 1:**
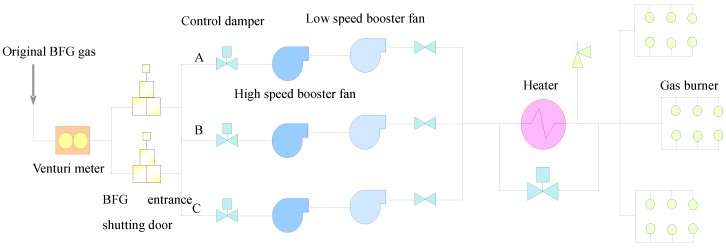
The structure of the BFG booster fans in Unit 4.

Blast furnace gas (BFG) is a byproduct of iron-making, whose production in iron-making increases from year to year due to the growth of the iron and steel industry. The blast furnace gas (BFG) is a recyclable energy gas and plays an important role in the energy consumption of iron and steel works. The power station in a steel-making plant would try to generate energy with the BFG. The Unit 4 studied in this work is the first 350 MW unit in the world which fully combusts the BFG. Its combustion ability can reach 1 million m^3^/h. The structure of the Unit 4 is displayed in Figure 1 [31]. Unit 4 is equipped with three dual-speed BFG booster fans. These fans are of importance and used to pressurize the BFG and make sure it can go into the furnace safely and combust stably. There is a tower-type once-through boiler in this unit, along with 18 compound gas burners that are well-distributed in three tiers. They are arranged separately on the front wall and back wall of the boiler.

The technological process of the BFG recovery unit is as follows:

The original BFG gas from the the Department of Energy and Environment Protection → Venturi meter → BFG entrance shutting door → BFG booster fan entrance control damper → three BFG booster fans → BFG booster fan exit isolation damper → Heater and its bypass → BFG gas burners.

The speed of the booster fans is divided into two levels: 744 r/min and 993 r/min, whose corresponding powers are 730 kW and 1650 kW, respectively.

### 2.2. Condition Data Acquisition Testing Framework 

For iron-making, lots of rotating machineries work in the complex process going from iron ore to steel products. The equipment is in long-term use under complex conditions. This may lead to various types of fault and cause huge economy losses. However, the key parts of rotating machinery are not stationary and therefore not easy to change, so it is crucial to carry out effective condition monitoring and fault diagnosis. 

For this purpose, a testing system focusing on extracting abnormal condition information from vibration signals is designed. This system works on the data acquisition level and network database level of the testing and diagnosis system in the steel-making plant. Off-line vibration tests are conducted for iron and steel smelting mechanical equipment using portable data acquisition devices such as the CSI2320, Telesens8823, SONY-EX and so on. These devices can selectively implement hardware integration for acquired signals to save the data.

**Figure 2:**
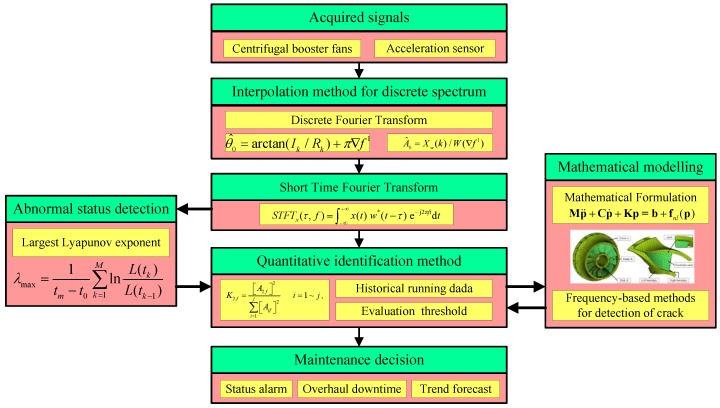
The work flow diagram of the research framework.

The feature information can be extracted from the vibrations by means of traditional spectrum analysis, characteristic spectrum analysis and special feature extraction modules in the testing system. The change trends of these features can be used to judge the working condition of the equipment and the appearance of incipient faults. The software interface of the testing system is implemented based on Labview 7.0. The testing system is programmed with a mixture of Labview and Visual Studio routines with consideration to execution efficiency. In addition, SQL Server is introduced as the extended interface to access the internal database. Moreover, simple tips about the main functions are available in a help module. The system also has other functions such as saving results, report generation, *etc*. The whole work flow diagram of the quantitative identification research framework is displayed in Figure 2.

### 2.3. Fault Feature Extraction Method

#### 2.3.1. Discrete Spectrum Interpolation Method 

The rotating frequency and its harmonic components can reflect the fault features concerning misalignment, rub-impact, and dynamic unbalance. These features are usually extracted from the frequency spectrum. However, the FFT and spectrum analysis would cause energy leakage owing to time domain truncation and the interference of noise [26,27]. This may lead to great errors in the frequency, amplitude and phase in the FFT and spectrum analysis. In order to improve the accuracy, a discrete spectrum interpolation method is adopted.

Let *x*(*t*) be a harmonic signal sequence with frequency *f*_0_, amplitude *A*_0_ and phase θ_0_. Suppose the amplitude and phase first calculated by a Discrete Fourier Transform (DFT) and then corrected by the interpolation method are:

(1) $${\hat{A}}_{0}=X_{w}(k)/W(\nabla f^{1})$$

(2) $${\hat{\theta }}_{0}=\arctan(I_{k}/R_{k})+\pi \nabla f^{1}$$

where $X_{w}(k)$ means the *kth* line of the harmonic signal, *i.e.*, the maximum value of the main lobe. *W*(∇*f*^1^) expresses the frequency spectrum for a rectangular window with the value *W*(∇*f*^1^) = sin(*π*∇*f*^1^)/ (*π*∇*f*^1^). *R_k_* and *I_k_* represent the real and imaginary parts of DFT, respectively. When rotating frequency is input, the system would correct the amplitude and phase of the rotating frequency and search for the accurate amplitude and phase of its harmonic components automatically.

#### 2.3.2. Short Time Fourier Transform

Blade crack fault diagnosis is a problem that troubles scholars and engineers at home and abroad. Online and offline condition monitoring systems are widely used in rotating machinery. The key problem is how to choose the method to process the signal from the industrial field and obtain the fault characteristics. The short-time Fourier transform (STFT) is one of the earliest and the most basic methods used for time-frequency analysis [28]. The STFT is one of the most widely used algorithms in signal processing and fault diagnosis based on a detailed Fourier transform centered at each time point. In STFT, the signal is compared with window functions that are concentrated in both the time and frequency domains. The STFT algorithm and the window function can be mathematically represented as follows:

(3) $$\begin{array}{ll} STFT_{x}(\tau ,\;f) & =\int_{-\infty }^{+\infty }x(t)\;w^{*}(t-\tau ){\;e}^{-j2\pi ft}dt\\ & =\int_{-\infty }^{+\infty }x(t){\left[w(t-\tau )e^{j\;2\pi ft}\right]}^{\;*}dt\\ & =\langle x(t),\;w(t-\tau )e^{j\;2\pi ft}\rangle \end{array}$$

where, *w*(*t*) is the window function which has a user defined time duration; and *x*(*t*) is the waveform signal in the time domain.

## 3. Case Study via Blade Crack Fault of a Centrifugal Booster Fan

**Figure 3:**
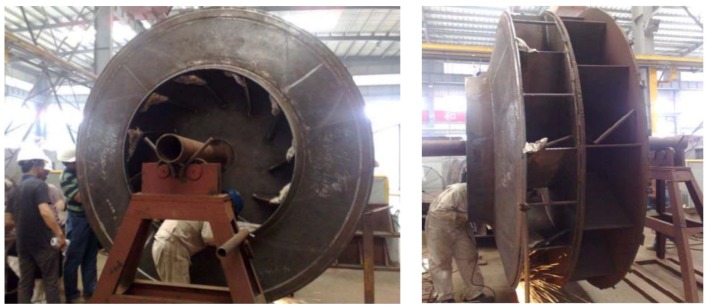
Photos of the rotor blades of No. A fan.

**Figure 4:**
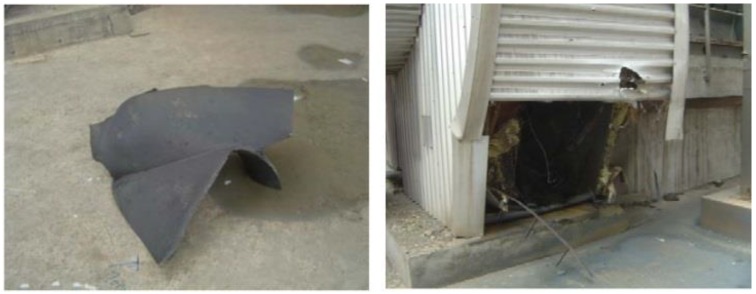
Site photos of the broken blade.

Unit 4 in a power station composed of three imported fans. As shown in Figure 3 [24,31,32], the rotor blades in No. A fan are welded on the entrance control damper. The three fans performed well since they were first used in production, and had never been overhauled before. On 20 July 2011, the rotor broke apart during the process of switching from low speed to high speed. Pieces of the blades flew out of the volute. Figures from the scene are shown in Figure 4 [24,31,32]. The bearing box in the drive end and the coupling are crushed. Besides, the main shaft is seriously deformed. By analysis of the causes that produced the accident, we find that there were blade cracks in the booster fans.

**Figure 5:**
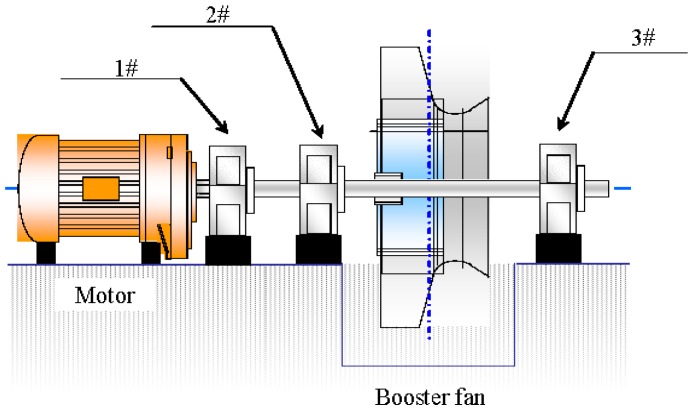
A sketch of the three groups of sensors.

In order to extract the vibration characteristics during crack growth and gain experience for condition monitoring on the same type of unit, the testing system as described before is used to analyze the historical data. From 4 August 2010 to 6 July 2011 (nearly a year before the accident), data acquisition of the two bearings, which support the rotor, was carried out by the industry technological service company at the sampling frequency of 2560 Hz with the length of 4096, ten times, including low speed (744 r/min) and high speed (993 r/min). Three groups of sensors are used, as shown in Figure 5. 

Two sensors are mounted on the two bearings. The vibration data from the horizontal, vertical and radial directions are obtained in each sensor. In addition, a sensor is mounted on the motor. The data from the vertical direction is obtained there. The details about the running status data acquisition are shown in Table 1, where D means low speed, Dh means high speed and GE means the amplitude of the envelope in g. A means radial direction, H indicates horizontal direction and V is the vertical direction.

**Figure 6:**
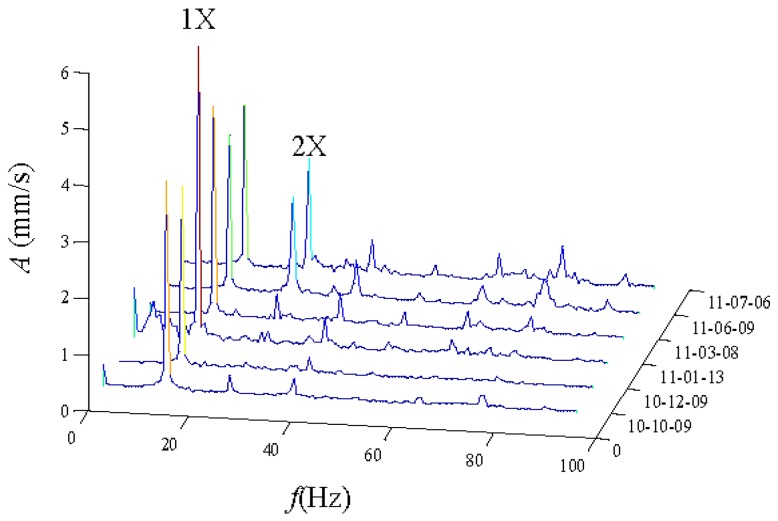
The FFT waterfall from 0 to 100 Hz of the No. 2H sensor.

The vibration data from the drive end in the horizontal direction is chosen for analysis. We can see that there is no a distinctive trend that would allow identifying crack faults. The waterfall plot based on FFT of No. 2H sensor data from 0 to 100 Hz is displayed in Figure 6 [23,24]. We can clearly observe the rotating frequency and its harmonic components. As time goes by, the amplitude of the rotating frequency increases first and then drops. However, the amplitude of the second harmonic drops first and then increases. From the point of view of the dynamics, the cracks close and open twice in one cycle of the rotation. The amplitude of the vibration response signal changes twice due to the change of the rotor stiffness from large to small, so the characteristics of the amplitude increase in the second harmonic could be used to indicate a blade crack.

**Figure 7:**
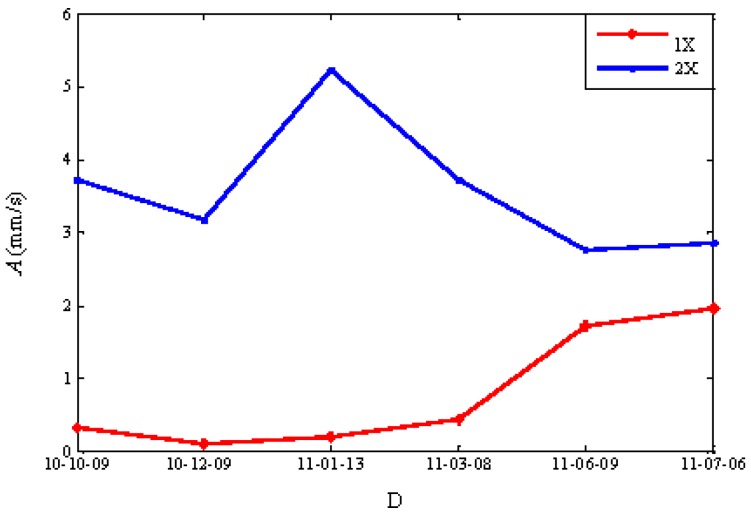
The trends of the amplitude value by the interpolation method for the discrete spectrum of the No. 2H sensor.

In order to obtain more accurate amplitude and phase data of the rotating frequency and the second harmonic component, a discrete spectrum interpolation method is used to analyze the signals of the No. 2 and No. 3 sensors in the horizontal direction. The result after correction is shown in Figure 7 [23,24]. As shown in Figure 7, the amplitude of the rotating frequency first increased and then decreased. The amplitude of the second harmonic component increases constantly. A breathing crack is considered to appear from January to March in 2011 according to the results. The rotor stiffness changes twice in every rotating cycle. This leads to the increase of the amplitude of the second harmonic component. 

**Figure 8:**
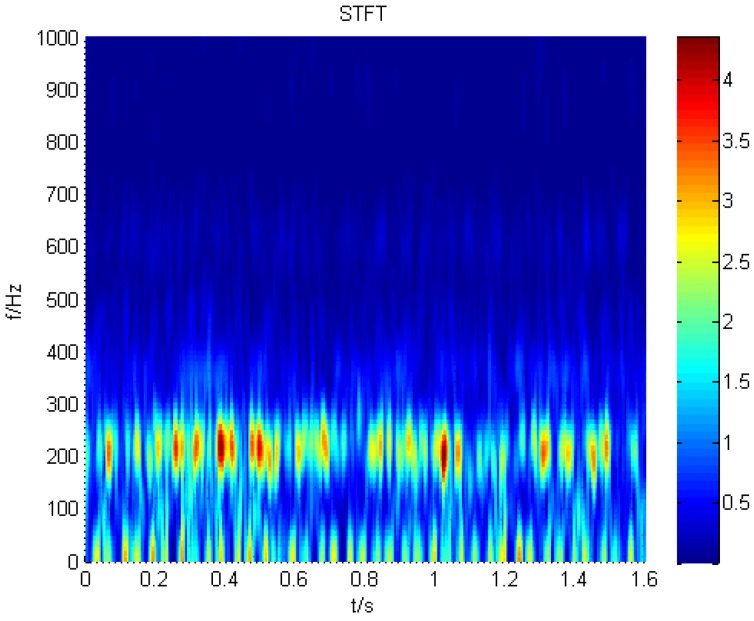
The STFT result acquired from a normal blade.

**Figure 9:**
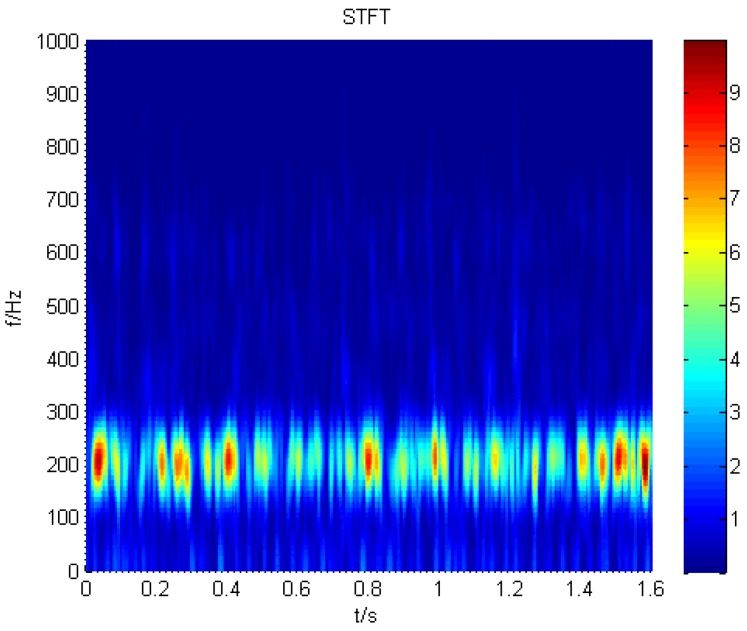
The STFT result acquired from a cracked blade.

For the purpose of extracting the non-stationary characteristics of the cracked rotor, STFT is employed to process the signal of the normal and cracked rotor. The result is displayed in Figure 8. From the figure, we can find a component that is obviously equal to the rotation frequency of the fan, which is 12.5 Hz. When cracks appear, there is a discontinuous line at the frequency of the second harmonic, which fluctuates at the rotating frequency and its second harmonic, as shown in Figure 9.

**Figure 10:**
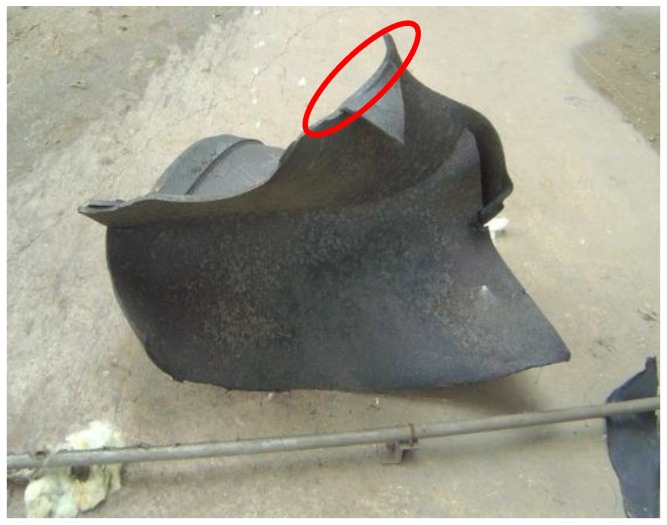
The photo of the cracked blade in the A fan.

The photo of the cracked rotor on the A fan is displayed in Figure 10. In this section, two methods are applied to analyze the blade crack fault. The vibration characteristics of the crack blade are extracted. This can help to improve the qualitative diagnosis performance for blade cracks, but the detection of crack damage based on the vibration signals is still less studied, and especially aquantitative identification method for scheduling reasonable maintenance plans is lacking.

## 4. Quantitative Identification and Abnormality Alarm Strategy for Blade Crack Faults

### 4.1. The Proposed Quantitative Identification Method 

Both Sun and Chen have attempted to propose a quantitative identification index for blade crack identification and have obtained certain achievements [24,31], but a more comprehensive abnormality alarm strategy via a quantitative identification index should be proposed to indicate the unbalancedness and implement necessary condition-based maintenance, so a quantitative identification method based on compound indexes including a traditional index and new index is developed in this section.

#### 4.1.1. Largest Lyapunov Exponent Algorithm

**Figure 11:**
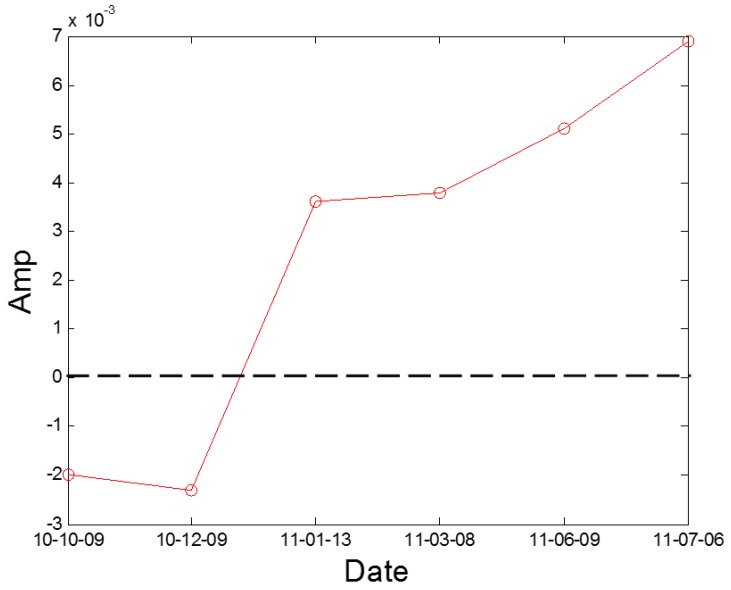
The LLE value of the A fan No. 2H sensor.

Lyapunov exponents, which measure the exponential rates of divergence or convergence of nearby trajectories in state space, are generally calculated to characterize chaotic processes. If the largest value in the spectrum of Lyapunov exponents is positive, it means that the system is chaotic. The largest value equal to zero indicates periodic or quasi-periodic dynamics. If all Lyapunov exponents are negative then the stable critical point is an attractor [29,30]. Among all the Lyapunov exponents, the Largest Lyapunov Exponent (LLE) has aroused considerable interest for its significant practical applications. The LLE has been applied to many fields for its notable capabilities. In this paper, the LLE is calculated as an indicator of the chaotic behavior of the load demand by using Wolf’s algorithm, which is given as [29,30]:

(4) $$\lambda_{\max}=\frac{1}{t_{m}-t_{0}}\sum_{k=1}^{M}\ln\frac{L\prime (t_{k})}{L(t_{k-1})}$$

where *L*’(*t_k_*) and *L*’(*t_k–_*_1_) mean the Euclidean distances computed between the nearest neighboring points on the different trajectories of the attractor at the *t_k_* and *t_k–_*_1_ time steps, respectively [29]. *m* indicates the number of replacement steps or iteration number. Details on the calculation parameter selection are given in [29,30]. The negative value of LLE indicates normal condition and a positive value of LLE indicates non-linear conditions, then the value of LLE can be used to initially identify the mechanical system state. According to the principle of the LLE algorithm, the LLE value of condition data from the No. 2H sensor is computed and displayed in Figure 11. We can observe that the non-linear condition appears after 9 December 2010. In addition, we still need a quantitative identification index to confirm the degree of crack fault.

#### 4.1.2. Relative Energy Ratio at Second Harmonic Frequency Component

According to the previous section, we know that when a breathing crack appears, the rotor stiffness changes twice from large to small in one rotation cycle [33,34]. The amplitude of the second harmonic increases obviously and the phase also changed significantly [35,36]. Hence, a quantitative identification method for blade crack fault and a new index are proposed to describe the degree of damage accurately when cracks grow. The new index is called relative energy ratio at second harmonic frequency component and expressed as *K*_2*f*_:

(5) $$K_{2f}=\frac{{\left[A_{2f}\right]}^{2}}{\sum_{i=1}^{j}{\left[A_{if}\right]}^{2}}\;i=1\sim j$$

where *A_f_* means the amplitude of the rotating frequency and *A*_2*f*_ means the amplitude of the second harmonic. Moreover, we can find that the energy of the first six order harmonic accounts for more than 98% of the total energy, so based on the amplitude of the frequency spectrum, the parameter *j* is selected as *j* = 6.

The value of *K*_2*f*_ can be used to judge the degree of the damage, where *a* and *b* are thresholds that need to be decided:

(1) 0 ≤ *K*_2*f*_ < *a* : Normal condition, the rotor works well;
(2) *a* ≤ *K*_2*f*_ ≤ *b*: Incipient fault, a crack appears, but is small;
(3) *K*_2*f*_ > *b*: Serious fault, many cracks appear.

**Figure 12:**
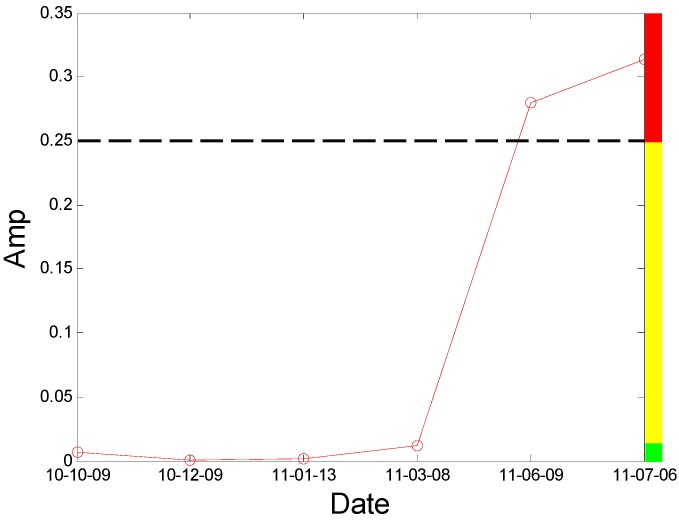
The results by the quantitative identification index on A fan No. 2H sensor.

During the period of August 2010 to December 2010, the rotor worked normally and we can think the reliability of this fan remained at the normal level of 0.9 during this time. The values of *K*_2*f*_ calculated from the bearing data in the horizontal direction are shown in Figure 12. According to the LLE value in Figure 11, a non-linear condition appeared after 9 December 2010. Moreover, according to the value of *K*_2*f*_ in December 2010 to March 2011 as shown in the Figure 12, the threshold value of *a* is preliminarily determined as 0.02. On 20 July 2011, the rotor broke apart during the process of switching from low speed to high speed (from 744 r/min to 993 r/min) and we can think that the reliability of this fan has decreased to 0 at this time. Based on the lowest reliability requirement the steel-making plant requires for safe running and the value of *K*_2*f*_ in bearing 2 from June 2011 to July 2011, the value of *b* is determined as 0.25 for safety’s sake and the reliability of this fan has decreased below 0.5 at this time by linear mapping relationship analysis, so the parameters are determined completely.

(6) $$\begin{array}{l} a=0.02\\ b=0.25 \end{array}$$

The new index *K*_2*f*_ is introduced into the testing system and works well in the condition monitoring of the centrifugal booster fan. The results in Figure 12 could be employed to analyze the condition of No. A fan.

**Figure 13:**
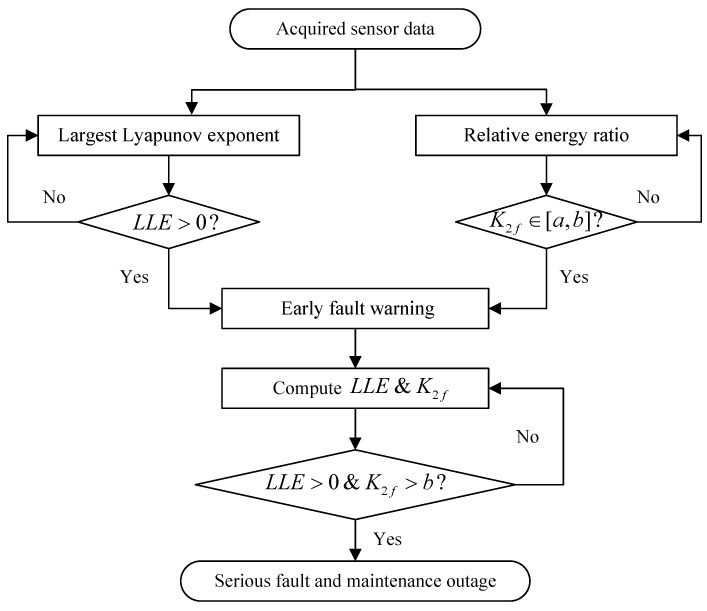
The flow chart of the proposed quantitative identification and abnormality alarm strategy.

Based on the proposed quantitative identification method, the abnormality alarm strategy can be obtained, and then the condition-based maintenance actions can be arranged reasonably to ensure safe and reliable operation. To sum up, the proposed quantitative identification and abnormality alarm strategy procedure of using sensor-dependent vibration data for blade crack identification in centrifugal booster fans can be summarized by the flow chart displayed in Figure 13. 

Meanwhile, the process of the proposed method and strategy for the mentioned engineering tasks in the power station can be summarized as follows:

(1) Collect the sensor-dependent running condition vibration data;
(2) Pre-process this vibration data using the discrete spectrum interpolation method and STFT;
(3) Compute the LLE and the relative energy ratio *K*_2*f*_.
(4) Confirm the degree of blade crack fault(s) of the centrifugal booster fan;
(5) Conduct the corresponding maintenance management activities based on the fault degree.

### 4.2. Test and Validation

**Figure 14:**
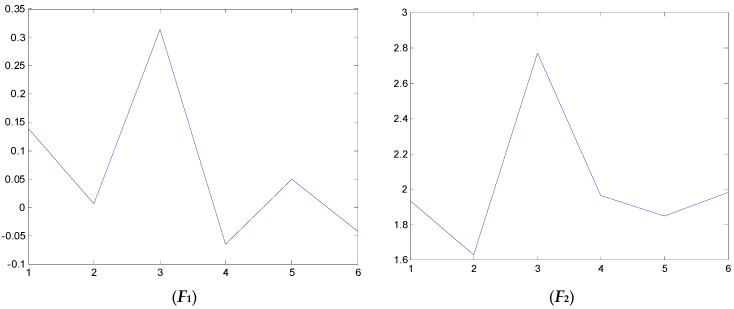

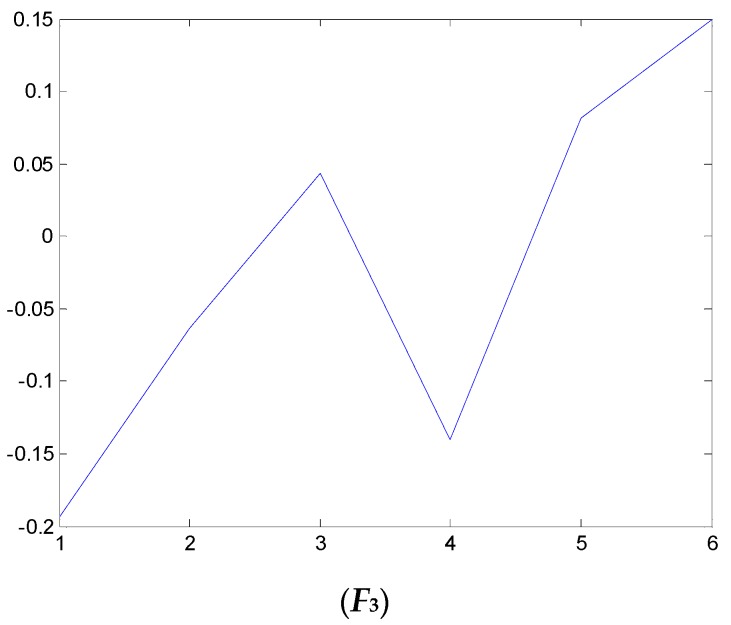
The analyzed results of A fan No. 2H sensor by ***F*_1_**, ***F*_2_** and ***F*_3_**.

**Figure 15:**
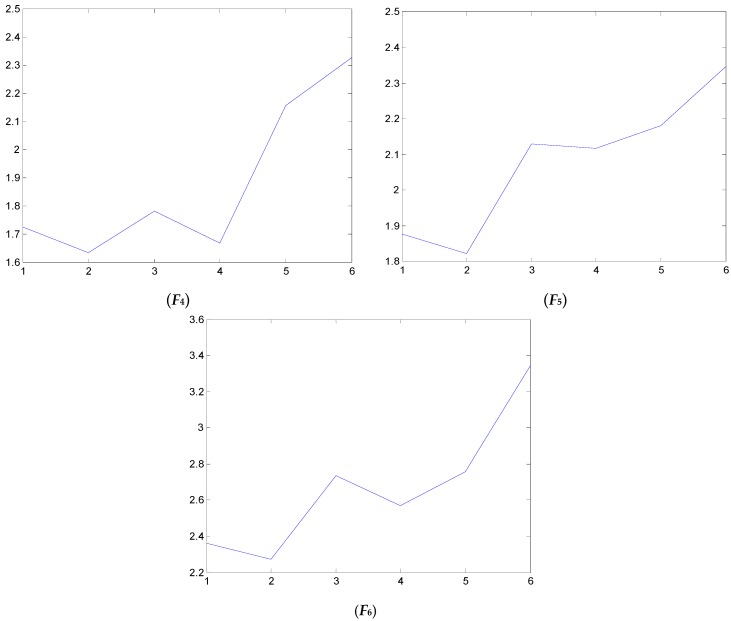
The analyzed results of A fan No. 2H sensor by ***F*_4_**, ***F*_5_** and ***F*_6_**.

**Figure 16:**
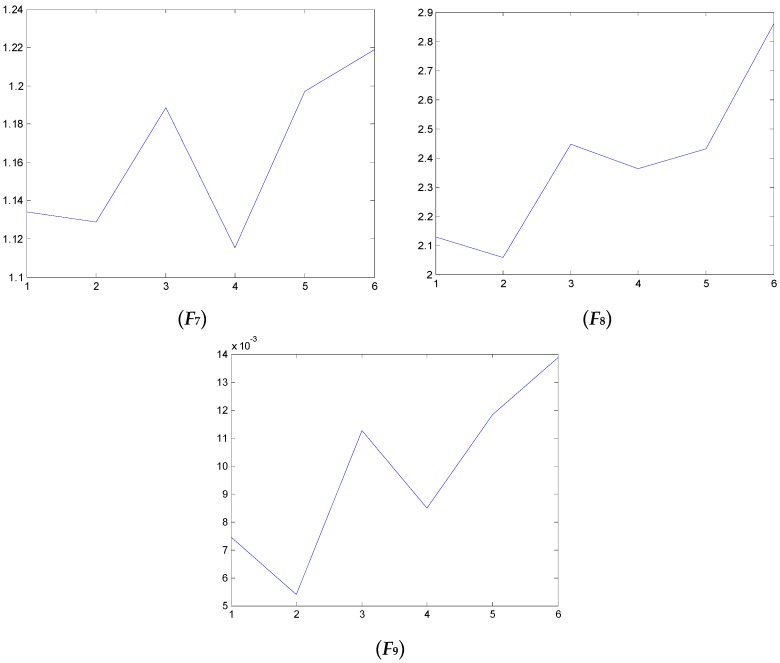
The analyzed results of A fan No. 2H sensor by ***F*_7_**, ***F*_8_** and ***F*_9_**.

**Figure 17:**
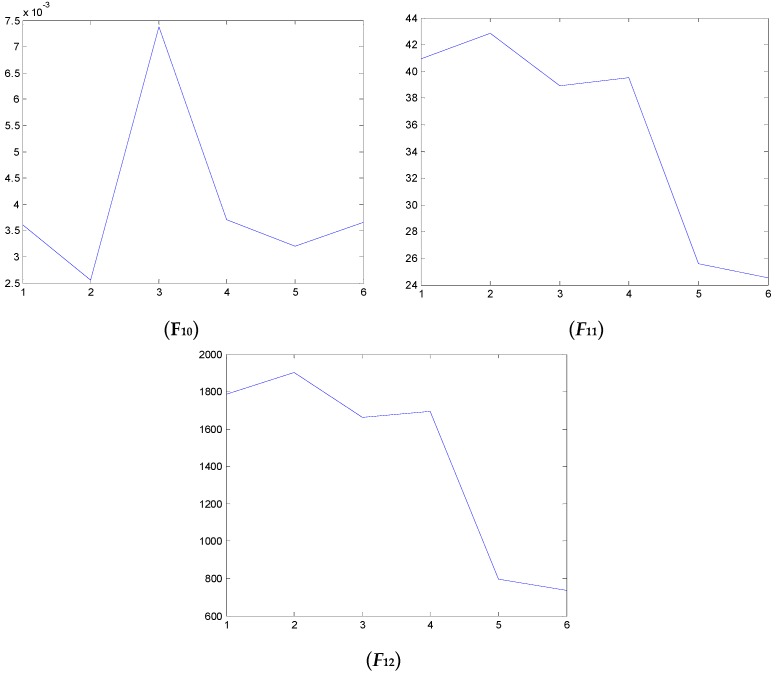
The analyzed results of A fan No. 2H sensor by ***F*_10_**, ***F*_11_** and ***F*_12_**.

**Figure 18:**
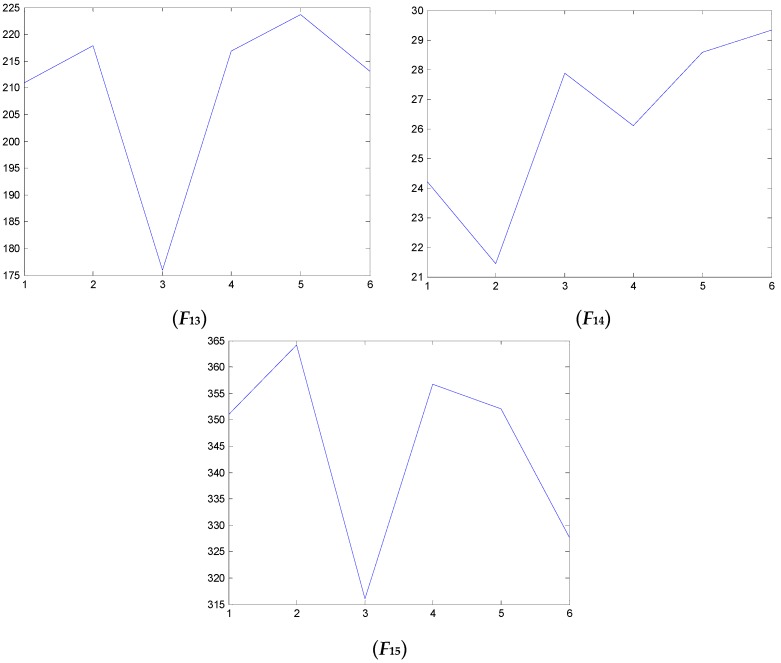
The analyzed results of A fan No. 2H sensor by ***F*_13_**, ***F*_14_** and ***F*_15_**.

**Figure 19:**
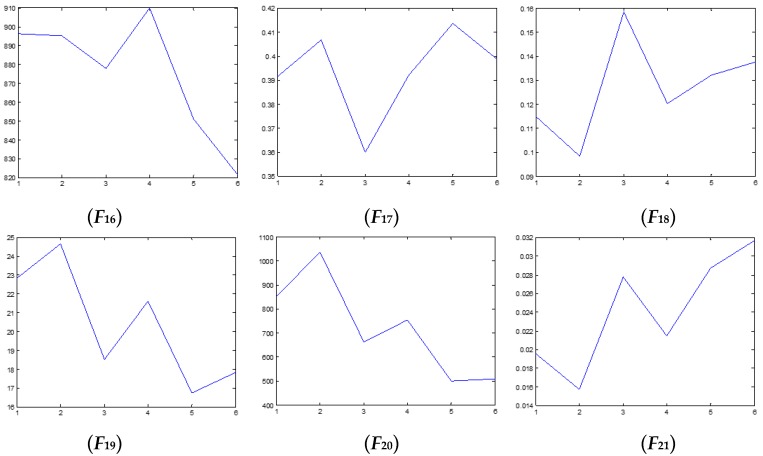
The analyzed results of A fan No. 2H sensor by ***F*_16_**–***F*_21_**.

We have also examined the performance of statistical features reported in the literature as comparisons to validate the performance of the proposed method. Some of the feature parameters have been demonstrated to be ineffective in previous publications, but in different papers, different feature parameters are applied according to the experience accumulated by different researchers. In the different applications, different feature parameters give different diagnosis performance. Thus, many feature parameters are calculated in this study. In total, 21 feature values are obtained, shown in Table 2. These features are adopted to indicate the faulty condition from the acquired vibration signals. The results are displayed in Figure 14, Figure 15, Figure 16, Figure 17, Figure 18 and Figure 19. According to the analyzed figures, some results can be obtained. First, obvious trends cannot be found the majority of the mentioned 21 feature parameters, except for the feature values *F*_4_, *F*_11_ and *F*_12_, so they are of no use for blade crack identification in centrifugal booster fans. Next, the feature values of *F*_4_, *F*_11_ and *F*_12_ show relatively clear trends compared to the remaining feature values, but the proposed strategy using compound feature parameters can indicate the running condition of centrifugal booster fans by hierarchical descriptions. The contrastive results demonstrate the effectiveness of the proposed strategy for the engineering task at hand.

**Figure 20:**
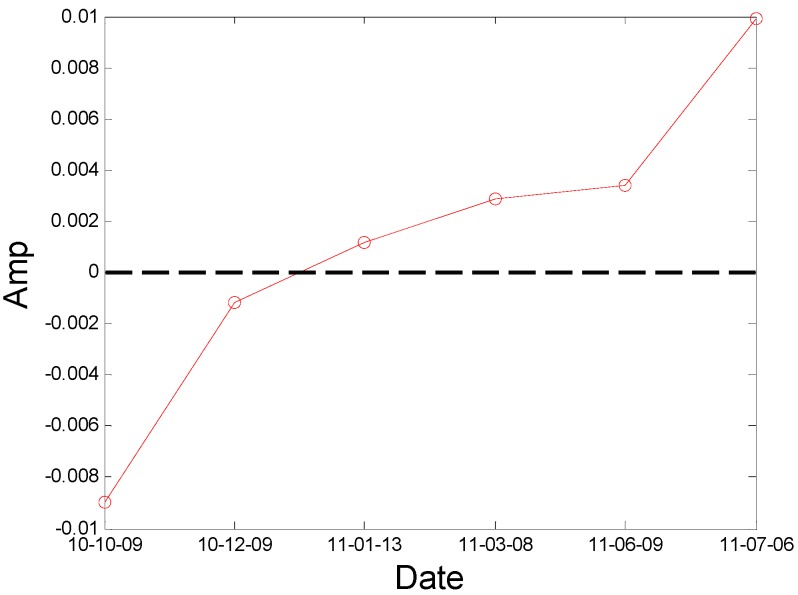
The LLE value of A fan No. 3H sensor.

**Figure 21:**
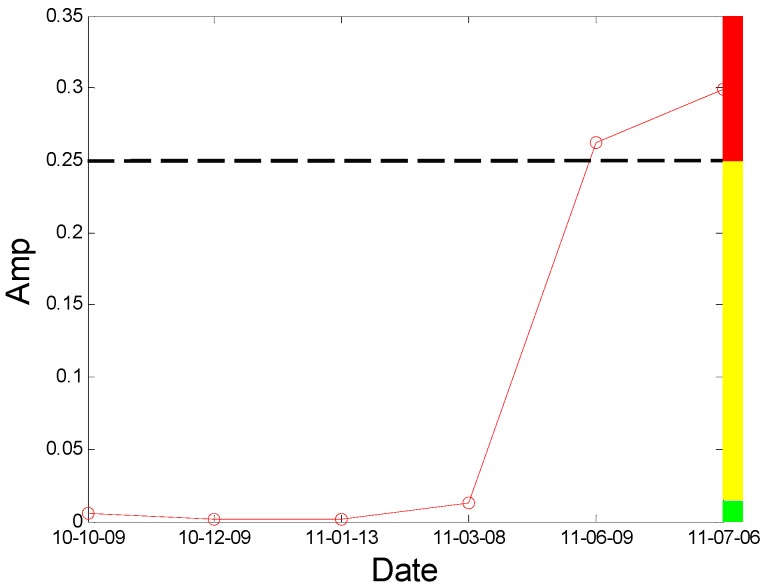
The result by the quantitative identification index of A fan No. 3H sensor.

Moreover, the proposed blade crack quantitative identification method is applied to identifying the running condition of No. A fan using A fan No. 3H sensor data. According to the principle of the Largest Lyapunov Exponent algorithm, the LLE value of the condition data from the No. 3H sensor is computed and displayed in Figure 20. We can observe that the non-linear condition also appears after 9 December 2010. In addition, the index *K*_2*f*_ is also used to identify the running condition of No. A fan using A fan No. 3H sensor data and the results are displayed in Figure 21. From the result, we also can obtain a clear trend to indicate the blade cracks in centrifugal booster fans and the value is above the abnormal warning stage after 8 March 2011. The result indicates that the proposed index and alarm strategy is feasible.

**Figure 22:**
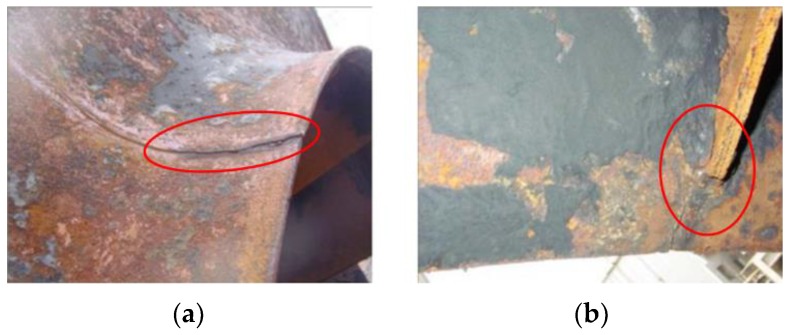
Photo of the crack on B fan.

**Figure 23:**
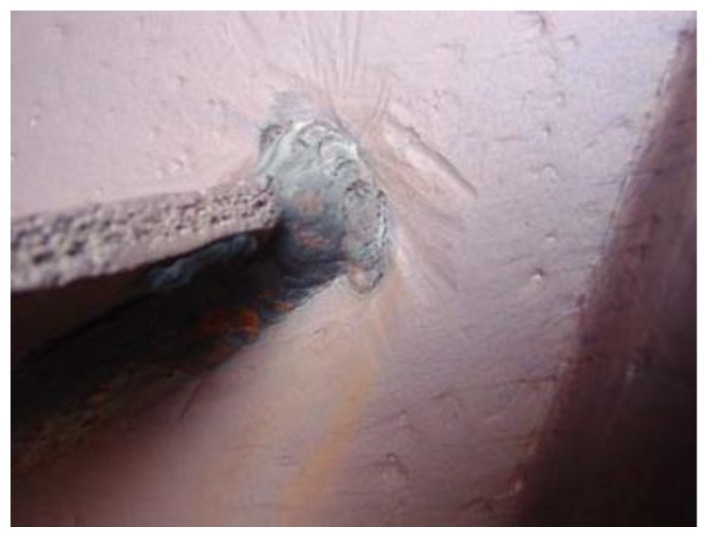
Photo of C fan after repair welding.

The proposed blade crack quantitative identification method is also applied to identifying the running condition of the remaining centrifugal booster fans. As mentioned previously, there are three fans in the unit 4. While No. A fan was being repaired, the quantitative identification index is also used to examine the No. B and No. C fans. These three fans have similar structures and functions, so the results based on the proposed method could be employed to analyze the condition of the No. B and No. C fan. After calculation and comparison, the values LLE > 0 and *K*_2*f*_ = 0.024 show that the No. C fan is in an incipient fault state. It should be focused on and chosen for monitoring. In addition, the fault status of the No. B fan (*K*_2*f*_ = 0.33) is much more serious. It needs to be repaired to avoid it breaking apart like No. A fan. During the checks, cracks were found in the No. B fan and No. C fan as expected. Figure 22 and Figure 23 [24] show the results. From the Figure 22a, we can see that the crack grows from the fan entrance and propagates along the radial direction of the damper. Figure 22b shows the welding position of the blade and the damper. Stress concentration in this position led to the growth and propagation of the crack. As shown in Figure 23, a crack appears at the welding position with a length of 3–4 cm in No. C fan. After detecting these cracks, the maintenance for No. B and No. C fans was carried out in March 2012 to prevent further accidents. These above results show the effectiveness and robustness of the proposed quantitative detection method for blade crack faults. 

## 5. Mathematical Modelling for Revealing Vibration Signal Properties

**Figure 24:**
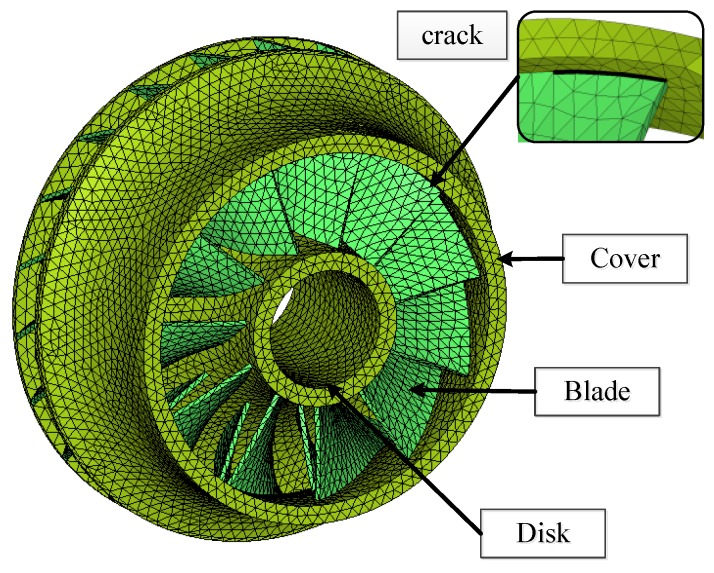
Finite element model of an impeller and a sector model.

An impeller consists of a cover component, a disk component and several blades. The finite element model of an impeller is depicted in Figure 24. 

According to many experienced engineers, cracks initiate mostly at the weld toe on the cover sides of the blade, as shown in Figure 24. In this paper, the effects of a crack located at such a position on the vibration response of the impeller are of interest, and the cracks at other position can be modeled without much modification. For details of the mathematical modelling and mathematical formulation readers may refer to [34,37].

After component synthesis, the equation of motion of a complete impeller is represented by:

(7) $$M\ddot{p}+C\dot{p}+Kp=b+f_{nl}(p)$$

where **M**, **C** and **K** are the mass, damping and stiffness matrices of the ROM; **p** is the vector of the DOFs retained; **b** denotes the external excitation acting on the impeller; and **f***_nl_*(**p**) is the nonlinear forces caused by intermittent contact of the crack surfaces. More details of this modelling process can be found in [34].

One of the most important issues for crack detection and identification is to search for sensitive indicators. An effective indicator should possess several features, such as robustness, monotonicity and industrial testability. The resonant frequencies discussed in the previous sections are potential indicators for the quantitative detection of crack faults. Two other kinds of frequency based indicators are studied in [34].

In sum, the frequency-based indicators for crack identification of centrifugal impellers were studied and discussed. However, an effective and reliable tool with a sensitive indicator for crack identification of impellers in operation still faces a lot of challenges at present.

## 6. Conclusions

In this paper, a vibration analysis method for the purpose of detection and quantitative identification of blade crack faults based on the amplitude of the rotating frequency is proposed. Aiming at the problem of energy leakage in FFT, a discrete spectrum interpolation method is proposed to extract the amplitude and phase accurately first. Then a quantitative identification and abnormality alarm strategy based on compound indexes including the Largest Lyapunov Exponent and relative energy ratio of the second harmonic frequency component is proposed. The results show that the proposed method is feasible. In the future, more effective signal processing methods should be studied and used to extract the characteristics of blade crack faults. Moreover, dynamic modeling and analysis of cracked rotor blades is necessary and urgent in future work. More reasonable effective indexes could be constructed to indicate the crack initiation and propagation from the point of view of dynamic analysis. Furthermore, although the proposed method shows good performance, more reasonable parameter selection for the terms *a* and *b* in *K*_2*f*_ should be studied based on plenty of running condition sensor-dependent vibration data in the future, and urgent demands, including quantitative diagnosis and fault location techniques, still remain to be established for scheduling reasonable maintenance plans.

## Figures and Tables

**Table 1:** The acquired running sensor-dependent vibration data.

No.	Date	2#			3#		
		A	H	V	A	H	V
		mm/s	mm/s	GE	mm/s	mm/s	GE
1	10-08-04	D	D	D	D	D	D
2	10-10-09	D	D	D	D	D	D
3	10-12-09	D	D	D	D	D	D
4	11-01-13	D	D	D	D	D	D
5	11-03-08	D	D	D	D	D	D
6	11-04-07	Dh	Dh	Dh	Dh	Dh	Dh
7	11-04-12	Dh	Dh	Dh	Dh	Dh	Dh
8	11-05-11	D	D	D	D	D	D
9	11-06-09	D	D	D	D	D	D
10	11-07-06	D	D	D	D	D	D

**Table 2:** The contrastive feature parameters.

Category	Parameter
Time-Domain index	$F_{1}=x\_m=\frac{\sum_{n=1}^{N}x(n)}{N}$ $x\_r={\left(\frac{\sum_{n=1}^{N}\sqrt{\left|x(n)\right|}}{N}\right)}^{2}$ $F_{2}=x\_std=\sqrt{\frac{\sum_{n=1}^{N}{\left(x(n)-F_{1}\right)}^{2}}{N-1}}$ x_peak=max|x(n)| x_pp=max(x(n))−min(x(n)) $F_{3}=x\_skew=\frac{\sum_{n=1}^{N}{\left(x(n)-x\_m\right)}^{3}}{(N-1)x\_std^{3}}$ $x\_rms=\sqrt{\frac{\sum_{n=1}^{N}{\left(x(n)\right)}^{2}}{N}}$ $x\_av=\frac{\sum_{n=1}^{N}\left|x(n)\right|}{N}$ $F_{4}=x\_kur=\frac{\sum_{n=1}^{N}{\left(x(n)-x\_m\right)}^{4}}{(N-1)x\_std^{4}}$ $F_{5}=x\_crest=\frac{x\_peak}{x\_rms}$ $F_{6}=x\_yu=\frac{x\_peak}{x\_r}$ $F_{7}=x\_shape=\frac{x\_rms}{x\_av}$ $F_{8}=x\_imp=\frac{x\_peak}{x\_av}$
Frequency-Domain index	$F_{9}=\frac{\sum_{k=1}^{K}s(k)}{K}$ $F_{10}=\frac{\sum_{k=1}^{K}{\left(s(k)-F_{9}\right)}^{2}}{K-1}$ $F_{11}=\frac{\sum_{k=1}^{K}{\left(s(k)-F_{9}\right)}^{3}}{K{\left(\sqrt{F_{10}}\right)}^{3}}$ $F_{12}=\frac{\sum_{k=1}^{K}{\left(s(k)-F_{9}\right)}^{4}}{KF_{10}^{2}}$ $F_{13}=\frac{\sum_{k=1}^{K}f_{k}s(k)}{\sum_{k=1}^{K}s(k)}$ $F_{14}=\sqrt{\frac{\sum_{k=1}^{K}{\left(f_{k}-F_{13}\right)}^{2}s(k)}{K}}$ $F_{15}=\sqrt{\frac{\sum_{k=1}^{K}f_{k}^{2}s(k)}{\sum_{k=1}^{K}s(k)}}$ $F_{16}=\sqrt{\frac{\sum_{k=1}^{K}f_{k}^{4}s(k)}{\sum_{k=1}^{K}f_{k}^{2}s(k)}}$ $F_{17}=\frac{\sum_{k=1}^{K}f_{k}^{2}s(k)}{\sqrt{\sum_{k=1}^{K}s(k)\sum_{k=1}^{K}f_{k}^{4}s(k)}}$ $F_{18}=\frac{F_{14}}{F_{13}}$ $F_{19}=\frac{\sum_{k=1}^{K}{\left(f_{k}-F_{13}\right)}^{3}s(k)}{KF_{14}^{3}}$ $F_{20}=\frac{\sum_{k=1}^{K}{\left(f_{k}-F1_{3}\right)}^{4}s(k)}{KF_{14}^{4}}$ $F_{21}=\frac{\sum_{k=1}^{K}{\left(f_{k}-F_{13}\right)}^{1/2}s(k)}{K\sqrt{F_{14}}}$

*x*(*n*) is the time domain signal, *n* = 1, 2, …, *N*; *N* is the sample point; *s*(*k*) is the spectrum of *x*(*n*), *k* = 1, 2, …, *K*; *K* is the number of spectrum lines; *f_K_* is the frequency of *k*-th spectrum line.
